# Less Is Fair: Reducing RTT Unfairness Through Buffer Sizing

**DOI:** 10.3390/s25175374

**Published:** 2025-09-01

**Authors:** Agnieszka Piotrowska

**Affiliations:** Department of Computer Networks and Systems, Silesian University of Technology, 44-100 Gliwice, Poland; agnieszka.piotrowska@polsl.pl

**Keywords:** BBR, Cubic, congestion control, buffer sizing, TCP, performance evaluation, RTT fairness

## Abstract

**Highlights:**

**What are the main findings?**

- Smaller buffers can substantially improve RTT fairness without degrading throughput.
- Bandwidth sharing is primarily governed by buffer size and RTT.

**What is the implication of the main finding?**

- Buffer sizing is not just about utilization, but fairness.
- Smarter buffer provisioning strategies can reduce the need for RTT-aware congestion control algorithms.

**Abstract:**

Sharing bottleneck bandwidth among TCP flows with diverse round-trip times (RTTs) remains a persistent challenge. This study investigates RTT unfairness and evaluates the behavior of two widely deployed congestion control algorithms, TCP Cubic and TCP BBR, under a variety of scenarios. The main objective is to better understand the underlying causes of RTT-based throughput disparity and to identify network configurations that promote fair bandwidth sharing. Using the Mininet emulation platform, extensive experiments were conducted to examine the effects of buffer size, sender distribution, and delay asymmetry on transmission performance metrics. The results show that while TCP BBR achieves high utilization with minimal buffering, its fairness depends on the interaction between RTT and buffer size. On the other hand, TCP Cubic achieves better fairness when moderate buffer sizes are provisioned and bandwidth imbalance is driven mostly by RTT ratio. These findings suggest that careful buffer sizing can reduce RTT unfairness and highlight the broader impact of queuing strategies on network performance.

## 1. Introduction

The first congestion control algorithm was introduced approximately 50 years ago [1]. Since then, dozens, if not hundreds, of variants have been developed, each with distinct design goals. Despite their differences, the fundamental objectives remain the same: maximizing bandwidth utilization, ensuring reliable data delivery, maintaining resilience under varying network conditions, and promoting fairness among competing flows. TCP, in particular, plays a critical role in safeguarding the Internet from congestion collapse and uncontrolled data flooding.

In 1979, Kleinrock proved that the optimal operating point for a network—one that ensures maximum throughput with minimal delay—is achieved when the volume of inflight data closely matches the Bandwidth-Delay Product (BDP) [2]. The BDP is defined as $RTT_{min}*BW_{max}$, where $BW_{max}$ denotes the available bottleneck bandwidth, and $RTT_{min}$ represents the minimum round-trip time (RTT) along the path, excluding any queuing delay. If inflight data falls below the BDP, the connection cannot fully utilize the available bandwidth. In contrast, exceeding the BDP leads to queue buildup and performance degradation.

Around the same time, Jaffe presented a paper showing that it was impossible to design a distributed algorithm that would converge at Kleinrock’s optimal operating point [3]. These results motivated research on alternative approaches to congestion control design. As a result, most traditional congestion control mechanisms adopted the loss-based approach, which inherently deviates from the optimal point by an amount proportional to the buffer size.

Loss-based TCP algorithms, including the widely deployed TCP Cubic [4], rely on packet loss as a primary signal of congestion, interpreting it as an indication that the network path is saturated. If routers permit the entire buffer to fill before initiating packet drops, Cubic tends to occupy all available buffer space. This behavior leads to substantial queuing delays, the magnitude of which is directly proportional to the buffer size. In the presence of large buffers, the queuing delay can exceed the base path RTT by an order of magnitude or more.

In contrast, TCP BBR (bottleneck bandwidth and round-trip propagation time), introduced by Google [5], seeks to approximate Kleinrock’s optimal point by balancing high utilization with low latency. BBR adopts a model-based, rate-controlled approach by estimating both the available bottleneck bandwidth and the minimum round-trip propagation delay of the transmission path to calculate the BDP. To account for delayed and aggregated acknowledgment packets (ACKs), BBR limits the volume of inflight data to a maximum of 2 BDP. Numerous studies have demonstrated that BBR is resilient to random losses and that its queue occupancy typically does not exceed 2 BDP, regardless of the available buffer size [6,7]. However, its bandwidth and delay estimates are fragile to transient queue dynamics, which, in heterogeneous RTT settings, may result in the domination of long-RTT flows.

One of the key design objectives of congestion control algorithms is fairness, typically defined as equitable bandwidth sharing among competing flows [8]. Both Cubic and BBR demonstrate reasonable fairness when flows sharing the same bottleneck use the same congestion control algorithm and experience similar round-trip times (RTTs). However, maintaining fairness becomes significantly more challenging when flows have heterogeneous RTTs, a common scenario in real-world networks. This issue, known as RTT unfairness, arises when flows with differing propagation delays compete for shared bandwidth, leading to disproportionate throughput allocations.

Fair bandwidth sharing in networks with heterogeneous RTTs is a well-known and long-standing issue. Most congestion control algorithms tend to favor short RTT flows [9], such a behavior is often justified by their typically shorter lifetimes. While Cubic exhibits this tendency, its performance under diverse network conditions, has been less thoroughly examined. BBR, on the other hand, is widely believed to favor long RTT flows, and much of the literature focuses on this aspect.

RTT unfairness, especially in the case of BBR, can be mitigated by implementing Active Queue Management (AQM). This effect has been demonstrated in several studies [10,11]. Since 2015, the IETF has recommended the inclusion of AQM in all routers [12], particularly due to the availability of algorithms such as PIE [13] and CoDel [14], which require minimal configuration and automatically adapt to varying network conditions. However, despite these advantages, the widespread deployment of AQM has not yet occurred. These algorithms effectively address the problem of bufferbloat, improve RTT fairness, reduce unnecessary delays, and allow for smaller buffer sizes.

The purpose of any Active Queue Management (AQM) algorithm is to control queue size to reduce its variability and queuing delay, while still preserving the ability to absorb traffic bursts. This becomes increasingly important as memory has become inexpensive, prompting router manufacturers to equip devices with large buffer capacities. However, as noted in [15], modern congestion control algorithms typically do not require large buffers; in most cases, a buffer size of 2 BDP is more than sufficient for the most widely used TCP variants. In this context, we investigate whether the use of shallow buffers impacts RTT fairness while still maintaining acceptable bandwidth utilization.

Deep buffers reduce packet drop probability but also significantly increase transmission delays. Reducing buffer size limits excessive inflight data and shortens packet delays, but excessively small buffers can cause frequent drops, increasing retransmission overhead and potentially reducing goodput, particularly in high-BDP networks where the link could carry more data than is successfully delivered. To avoid bandwidth underutilization, buffer sizes should be sufficient to accommodate the inflight data required to saturate the link, taking into account the network BDP, flow RTTs, and congestion control dynamics. Within this range, shallow buffers improve RTT fairness without significantly degrading overall link utilization. This paper investigates the operating conditions for Cubic and BBR under these constraints.

This study undertakes the problem of mitigating RTT unfairness by using small buffers. While using AQM have been shown to significantly improve fairness among competing flows, it is hypothesized that shallow buffers alone may also contribute positively to this objective. To verify this hypothesis, two widely used TCP congestion control algorithms, Cubic and BBR, are evaluated in terms of how they allocate bandwidth across flows with heterogeneous RTTs under varying buffer sizes. The main goal is to identify the conditions that lead to bandwidth disparities and determine the network configurations necessary to achieve fair distribution. Some of the findings have previously been demonstrated theoretically; here, they are revisited and validated experimentally within in a broader context. All experiments were conducted using the Mininet emulation platform.

The main contributions are as follows:

- The impact of buffer size, sender group composition, and RTT asymmetry on transmission performance is evaluated for both TCP Cubic and TCP BBR, focusing on throughput, retransmission behavior, and different aspects of fairness.
- The key parameters driving the unfairness in TCP Cubic and BBR were identified. We show how interaction between buffer size, RTT, and sender distribution shapes intra-group and inter-group bandwidth distribution.
- A detailed examination of how buffer size influences BBR congestion control behavior is provided, with emphasis on its bandwidth estimation mechanism. The study reveals that BBR’s fairness is governed by the interaction between buffer size and RTT.
- Contrary to prevailing assumptions, we show that RTT ratio is not a reliable predictor of degree of unfairness in BBR. Instead, fairness is primarily determined by whether the buffer is large enough to accommodate the flow’s congestion window gain, given its RTT.

The remainder of the paper is organized as follows. Section 2 reviews related work and provides a detailed discussion of the RTT fairness problem. Section 3 describes the methodology and the simulation framework. Section 4 presents the experimental results along with a discussion of the results. Finally, Section 5 concludes the paper.

## 2. Background and Related Work

Cubic [4] has been widely adopted as the default congestion control algorithm in major operating systems, including Linux, Windows, and MacOS, making it the most widely deployed TCP variant to date [16]. It also serves as the foundation for congestion control mechanisms in protocols such as QUIC [17] and the Stream Control Transmission Protocol (SCTP) [18]. A core design principle of Cubic is that flows with different round-trip times (RTTs) should achieve throughput ratios inversely proportional to their RTTs, implying that similar congestion window sizes are maintained regardless of path delay. This approach is further supported in [16], where it is argued that flows with differing RTTs should not necessarily receive equal throughput, and that a linear throughput-to-RTT ratio represents a more reasonable objective.

To provide a theoretical basis for analyzing this assumption, we refer to the fluid model framework introduced in [9], which describes the evolution of the TCP connection window under varying RTTs. Although designed to be independent of specific TCP variants, the model relies on key assumptions: all connections experience packet loss upon buffer saturation and respond to such losses accordingly. The author concludes that the influence of RTT heterogeneity depends strongly on buffer size relative to the BDP. With small buffers (below the minimum BDP of active flows), short-RTT flows dominate because their larger inverse RTT values drive faster window growth. Conversely, with larger buffers (above the mean BDP), performance becomes more sensitive to the number of long-RTT flows, as their slower feedback cycles amplify bandwidth disparities. These results hold within the assumptions of the fluid model of loss-based congestion control, synchronized losses, and absence of AQM and highlight how buffer sizing shapes the relative dominance of short- versus long-RTT flows. In this paper, this hypothesis is evaluated for TCP Cubic. The model, however, is not applicable to BBR, as its design does not conform to the loss-based assumptions underlying the fluid model.

RTT unfairness in Cubic cannot be attributed solely to the ACK-clocking mechanism, as was the case in earlier TCP variants such as Reno. Cubic was explicitly designed with a time-based cubic window growth function to reduce RTT bias, under the expectation that window evolution would be largely independent of acknowledgment pacing. Nevertheless, Cubic’s operation remains partly tied to ACK-clocking, since acknowledgments trigger actual window increases. As a result, shorter RTT flows still react faster to congestion feedback and can dominate bandwidth allocation. Further, Cubic’s fairness limitations have been linked to the role of the *K* parameter, which determines the time to the inflection point ($W_{max}$). Flows with larger RTTs have correspondingly larger *K* values, meaning they may fail to reach $W_{max}$ before the next congestion event, while shorter RTT flows may already be growing beyond it. This asymmetry leads to repeated reductions in the congestion window of long-RTT flows, reinforcing their disadvantage.

This behavior has been widely observed and documented in the literature, confirming that Cubic favors short-RTT flows [19,20], and showing that while Cubic mitigates some RTT bias relative to Reno, it does not fully eliminate it. RTT unfairness in Cubic cannot be fully mitigated through buffer size reduction alone, though smaller buffers may influence its severity and are, therefore, worth examining. Additionally, we investigate whether the often-assumed linear throughput-to-RTT ratio holds under heterogeneous RTTs and varying flow distributions.

BBR exhibits the opposite behavior compared to loss-based algorithms—it tends to favor long RTT flows [7]. This behavior raises concerns, as it can be exploited by artificially delaying acknowledgments to gain a disproportionate share of bandwidth. BBR’s effectiveness relies on accurate estimation of both the available bandwidth and the minimum RTT. A built-in synchronization mechanism is intended to align all flows during the Probe RTT phase, thereby improving the likelihood of correctly identifying the minimum RTT. However, deep buffers may disrupt this synchronization and, in some cases, delay the estimation. Nevertheless, the primary issue lies in the accurate estimation of available bandwidth, which is highly sensitive to buffer size.

BBR estimates the available bandwidth using the following formula:

(1) $$BW_{est}=\frac{Delivered\;data}{Measured\;RTT\;interval}.$$

BBR estimates available bandwidth based on the delivery rate observed during recent RTT samples in the ProbeBW phase. It maintains a windowed maximum bandwidth estimate (maxBW) over a default window of 10 RTTs. This persistence mechanism means that a single inflated sample, e.g., caused by ACK aggregation, can dominate the bandwidth estimate for an extended period, with the effect being more pronounced for longer RTT flows. This estimate is then used both to pace packets near the maximum delivery rate and to compute the BDP. The BDP, derived using a windowed minimum RTT estimate (updated at least once every 10 s), determines the cap on inflight data, limited to 2 BDP. Because the estimation is ACK-clocked, longer RTT flows update more slowly and may retain an artificially high maxBW value, which amplifies their throughput advantage and exacerbates RTT unfairness.

Overestimation of bandwidth leads to excessive inflight data, which short RTT flows are comparatively less able to absorb due to earlier congestion window limits. In shallow buffer scenarios, this excess traffic contributes to high queue occupancy and elevated retransmission rates. With deep buffers, the surplus data is absorbed, but tends to benefit longer RTT flows more. These flows inject more data, build larger queue shares, and receive ACKs in compressed bursts, which can further inflate their delivery rates and estimates—creating a self-reinforcing feedback loop. As the queueing delay grows beyond the minimum RTT, flows become congestion window-limited, particularly shorter RTT flows, which are capped earlier and are less able to utilize additional available bandwidth. Thus, queue dynamics and buffer size significantly influence fairness and bandwidth allocation in BBR.

The traditional BDP rule for buffer size is suitable for scenarios involving a single flow that halves its congestion window in response to packet loss. However, modern congestion control algorithms employ different rate reduction strategies. According to [15], Cubic requires a buffer size of approximately 3/7 BDP to achieve full link utilization, while BBR operates efficiently with buffers as small as 1/4 BDP. The same study also suggests that for multiple flows, the buffer size can still be scaled by $BDP/\sqrt{n}$ [21], although the authors acknowledge that this guideline may be overly conservative. Additional performance factors, such as goodput and retransmission ratio, should also be taken into account when determining appropriate buffer sizes.

The use of reduced buffer sizes to address known performance issues is well established. In [22], authors claim that smaller buffers may lead to bandwidth underutilization and an elevated retransmission rate. However, several studies demonstrate that buffer sizes can be reduced to a few dozen packets, provided that a slight reduction in link utilization is acceptable [23,24].

The influence of buffer size on BBR’s behavior has been analyzed in several studies [7,10,25]. A consistent conclusion emerges: once a persistent queue is established, a flow’s throughput is largely determined by its share of the queue. As buffer size increases, longer RTT flows—having larger BDP and, thus, higher inflight data caps—inject more data, leading to disproportionate queue occupancy and bandwidth unfairness. In contrast, with shallow buffers, flows are not significantly limited by their congestion windows, and RTT differences have a much smaller impact on bandwidth sharing.

In [26], the authors claim that BBR is well suited for networks with shallow buffers despite its high retransmission ratio, whereas loss-based algorithms perform better in deep buffer scenarios. They also demonstrate that, despite a much higher drop ratio, BBR maintains goodput better than Cubic in shallow buffers.

In 2019, Google introduced the second iteration of BBR (BBRv2) [27]. This version was designed to adapt more rapidly and improve convergence toward fair bandwidth sharing by reserving headroom for both loss-based and newly arriving flows. Unlike its predecessor, BBRv2 explicitly monitors packet losses and ECN signals to prevent excessive loss ratios, thereby addressing the problem of high retransmission rates. Empirical evaluations confirmed that BBRv2 better controls the volume of inflight data and substantially reduces packet losses and retransmissions compared to BBRv1 [11,28]. However, despite these improvements, BBRv2 still fails to achieve fair bandwidth sharing in the presence of heterogeneous RTTs or when competing with loss-based TCP variants.

The most recent iteration, BBRv3 [29], was intended to enhance convergence among BBR flows, coexist more effectively with loss-based congestion controls, and reduce queuing delays across buffer regimes. Yet, early evaluations contradict its design goals: BBRv3 shows disappointing performance compared to BBRv1, particularly in intra-fairness scenarios with flows of different RTTs, where RTT unfairness remains unresolved [10,30].

Numerous studies have addressed the RTT unfairness exhibited by BBR. Proposed improvements can be broadly categorized into three main approaches. The first group focuses on modifying pacing gain dynamics and the duration of the probing phase [31,32,33,34,35]. The second category introduces enhancements to bandwidth estimation and adjustments to the sending rate [36,37,38]. The third group involves modifications to congestion window gains, thereby altering the maximum inflight data allowed per flow [25,39,40].

In [31], the authors argue that short RTT flows are consistently disadvantaged in typical networks with persistent queues, irrespective of bandwidth, AQM implementation, RTT disparity, or the number of competing flows. To address this issue, they propose BBQ, a BBR variant that enforces uniformly shorter bandwidth probing intervals across all flows. By decoupling the probing duration from RTT, BBQ enables all flows to benefit equally from timely feedback, rather than favoring only those with shorter RTTs. In contrast, our study shows that the dominance of long RTT flows is not universal: under certain network configurations, such as shallow buffers, RTT differences have a smaller impact and short RTT flows are not necessarily disadvantaged.

In [32], the authors introduced Adaptive-BBR, which eliminates the fixed 8-cycle ProbeBW phase and dynamically adjusts the pacing gain to regulate the sending rate. BBR-GC [33] applies a gamma correction function to modify the pacing gains during the ProbeBW and Drain phases, allowing flow-specific rate adaptation and enhancing RTT fairness. Yang et al. [34] further refined this approach by proposing an enhanced pacing gain model grounded in BBR’s bandwidth estimation process. BBR-R [35] extends this line of work by refining delay detection: it allows flows to enter the ProbeRTT phase immediately when three consecutive RTT samples exceed a multiple of the minimum RTT, rather than waiting for the default 10 s. This ensures earlier queue drainage and more timely detection of persistent delay, while enforcing a minimum ProbeBW duration for stability.

Numerous studies have proposed enhancements to BBR to address bandwidth overestimation in large buffer scenarios. In [36], the authors introduced Delay-Aware BBR (DA-BBR), which incorporates a correction factor to adjust the delivery rate estimation for long RTT flows. DA-BBR reduces excessive inflight data and adapts the sending rate per flow, applying stronger reductions for longer RTTs. Zheng et al. [37] proposed a modification that dynamically adjusts the sending rate, preserves the ability to explore available bandwidth, and limits excessive data injection. Ad-BBR [38] replaced the static RTprop value with a dynamic new RTT (nRTT) estimate that reflects real-time link conditions. It then applies finer-grained, RTT-aware transmission rate adjustments through adaptive scaling factors, reducing bandwidth overestimation.

Another approach to controlling queue buildup by reducing the congestion window gain below two is presented in [39]. A similar approach, BBR-ACW, proposed in [25], dynamically adjusts each flow’s congestion window gain based on the delivery rate and estimated queue status. BBR-EFRA [40] extends this strategy by explicitly incorporating buffer queue status into both pacing rate and congestion window calculations. It adaptively reduces pacing during overload to drain excess inflight data, while adjusting each flow’s effective BDP to prevent persistent queue buildup and improve fairness.

Preventing bandwidth overestimation and reducing excessive inflight data in BBR has been shown to significantly lower retransmission rates, as emphasized in the aforementioned studies. While these enhancements improve RTT fairness, it is important to recognize the original rationale behind BBR’s congestion window gain of two, as set by the Google development team. This value was chosen to account for ACK delay and aggregation effects, which are common in real-world networks. Reducing the inflight limit too aggressively to match the ideal bottleneck share may lead to transmission stalling, particularly in practical deployments where delayed or batched ACKs reduce feedback granularity.

Tao et al. [41] developed a theoretical model analyzing BBR dynamics, demonstrating that bandwidth sharing is primarily determined by the RTT ratio, independent of initial sending rates and link capacity. This paper verifies those assumptions and extends the analysis to scenarios with multiple flows.

Existing studies on RTT unfairness primarily focus on either protocol-level modifications (e.g., pacing gains, bandwidth estimation, or congestion window adjustments) or network-level mechanisms such as AQM. Although these approaches demonstrate measurable improvements, they typically require changes at the end-host or router level, limiting their practicality and widespread adoption. Furthermore, most prior work has analyzed fairness only between pairs of flows with differing RTTs, overlooking more complex traffic scenarios. What remains underexplored is the extent to which buffer sizing alone, without modifying TCP implementations, can mitigate RTT unfairness in both Cubic and BBR across diverse flow distributions and RTT ratios, offering a lightweight and easily deployable solution. The following section outlines the experimental methodology and evaluation framework used in this study.

## 3. Experimental Setup and Metrics

**Figure 1:**
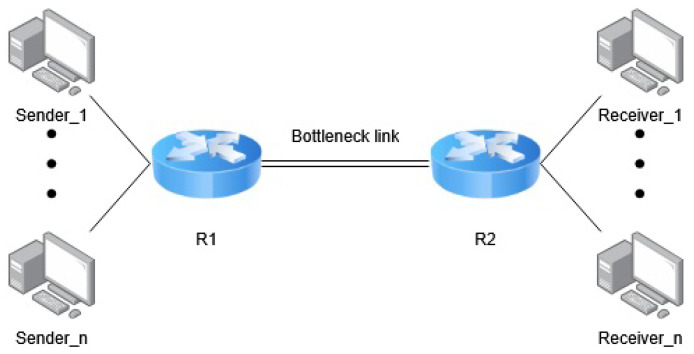
Dumbbell topology used for experiments.

To evaluate the impact of buffer size on RTT unfairness in congestion control algorithms, we conducted a series of experiments using Mininet. The network topology emulated a standard dumbbell configuration with two routers (see Figure 1). The analysis focused on comparing the performance of TCP Cubic and TCP BBR under varying buffer sizes, heterogeneous round-trip times (RTTs), and differing numbers of sender–receiver pairs, each communicating over distinct delay paths.

The number of senders and the corresponding receivers varied between scenarios. A dumbbell topology was employed, in which two sets of hosts communicate through a shared bottleneck link. This configuration enables controlled emulation of network congestion and flow competition. The topology remained consistent throughout all experiments. Router R1 was used to emulate propagation delays using NetEm [42]. The default queuing discipline on both routers was DropTail (DT). To simulate buffer constraints at the bottleneck, the queue size on router R1 was limited using Linux Traffic Control (tc) [43]. Scenario-specific configurations and parameters are detailed in the respective analysis sections.

Traffic between sender and receiver hosts was generated using iperf3 [44], which enabled the collection of key performance metrics, including throughput, retransmission ratio, and average RTT as measured at the sender. All flows were configured as continuous TCP streams, to ensure steady-state behavior and consistent measurement of congestion control dynamics. UDP traffic was not used. Queue occupancy on the interface connected to the bottleneck link was monitored periodically using Linux’s Traffic Control (tc) utility. For BBR-specific metrics, such as estimated bottleneck bandwidth, minimum RTT, and current RTT, data were periodically collected using the Linux ss command [45]. The packet size was set to 1500 B, with a segment size of 1460 B. The retransmission ratio was calculated as the number of retransmitted packets divided by the total number of transmitted (i.e., retransmitted and successfully received packets), and is reported as a percentage.

The bandwidth utilization was calculated as the sum of the flows’ throughputs divided by the link capacity and presented in percentages. The fairness metrics were calculated using the Jain’s fairness index as described in RFC 5166 [46]:

(2) $$JI=\frac{{\left(\sum_{i=1}^{n}x_{i}\right)}^{2}}{n\cdot \sum_{i=1}^{n}x_{i}^{2}},$$

where *n* is the number of active flows and $x_{i}$ is the throughput of the i−th flow, i=1,2,…,n. To evaluate the fairness of bandwidth sharing between flows with differing RTTs, the Symmetric Throughput Difference Ratio (STDR) was calculated, defined as:

(3) $$STDR=\frac{thr_{1}-thr_{2}}{thr_{1}+thr_{2}},$$

where $thr_{1}$ and $thr_{2}$ are the average throughput of flow 1 with a fixed RTT and flow 2, respectively.

**Figure 2:**
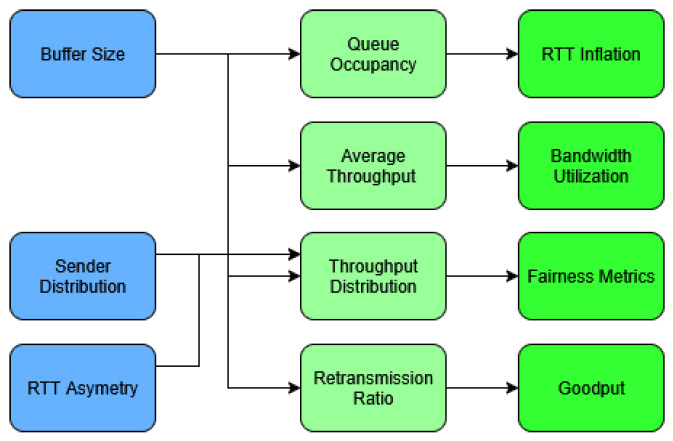
Conceptual relationships between measured performance metrics.

To provide an overview of the experimental framework, Figure 2 illustrates how the independent variables (buffer size, RTT asymmetry, and sender distribution) influence measured performance metrics (queue occupancy, throughput, and retransmission ratio), which are then used to derive higher-level indicators such as fairness metrics, RTT inflation, bandwidth utilization, and goodput.

Each configuration was executed for 100 s, which is a widely used duration in TCP performance studies. This length is sufficient for both Cubic and BBR to exit slow start and operate in steady state, as confirmed by stability in throughput and queue occupancy time series. To ensure statistical reliability, each experiment was repeated five times, and the results were averaged. The network topology was implemented using Mininet. All experiments were conducted on a dedicated server running Ubuntu 20.04 with kernel version 5.4.0-169-generic. The server was provisioned with sufficient computational resources to ensure that CPU and memory were not bottlenecks during the emulation. Specifically, the virtual machine was allocated 16 vCPUs operating at 2.30 GHz and 64 GB of RAM.

The simulation setup was developed using and extending scripts made available by [10]. All data and scripts used to obtain these results are publicly available online for reproducibility [47].

## 4. Results and Discussion

This section presents the results and analysis of TCP Cubic and TCP BBR performance under varying network conditions. Two experimental scenarios were evaluated: one involving two concurrent flows, and another with ten simultaneous flows.

### 4.1. Modeling RTT Fairness: Two-Flow Scenario

The scenario consists of two senders and two corresponding receivers. During the simulation, two flows are initiated simultaneously, each running for 100 s. The round-trip time (RTT) of the first flow is fixed at 20 ms, serving as the baseline RTT, while the RTT of the second flow is varied across 24 different values, ranging from 1 ms to 200 ms. The buffer size is defined as a percentage of the bandwidth-delay product (BDP), calculated based on the baseline RTT, and spans 32 different configurations from 0.01 BDP to 50 BDP. The link capacity for all experiments is set to 1 Gbps.

#### 4.1.1. Bandwidth Utilization

**Figure 3:**
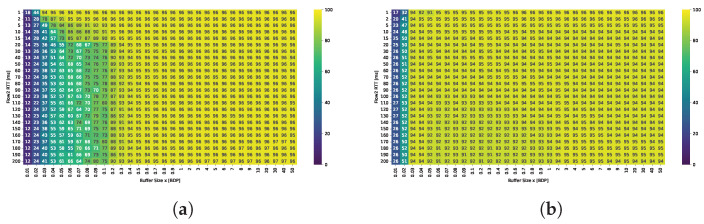
Comparison of bandwidth utilization [%] for (**a**) TCP Cubic and (**b**)TCP BBR under varying buffer sizes and RTTs.

The ability of both protocols to fully utilize the available bandwidth is evaluated under varying buffer sizes and RTTs of the second flow. Detailed results are gathered in Figure 3.

The results indicate that TCP BBR is capable of nearly fully utilizing the available bandwidth, even with minimal buffer sizes. Utilization dropped below 50% only for two smallest buffer values within the examined range, which were capable of holding no more than a dozen packets. TCP Cubic fails to achieve satisfactory bandwidth utilization unless the buffer size is at least 0.3 BDP. Under smaller buffer configurations, Cubic utilizes the available bandwidth effectively only when the RTT of the active flows is very low. For example, with a buffer of just 0.03 BDP, high bandwidth utilization was observed only when the flows had RTTs no greater than 2 ms; for these flows, the relative buffer size was 0.3 BDP.

#### 4.1.2. Throughput and Fairness

**Figure 4:**
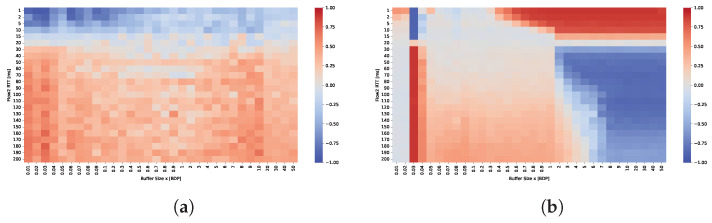
Comparison of STDR for (**a**) TCP Cubic and (**b**) TCP BBR under varying buffer sizes and RTTs.

Figure 4 presents the Symmetric Throughput Difference Ratio (STDR). Values approaching 1 (shown in red) indicate dominance of the fixed 20 ms RTT flow, while values near −1 (shown in blue) indicate dominance of the flow with varying RTT. White regions represent scenarios where the throughput difference between the two flows is negligible.

The results indicate that TCP Cubic achieves acceptable bandwidth sharing between the two flows only when the RTT difference is small. RTT fairness appears to depend primarily on the RTT ratio rather than on buffer size. However, the best fairness is observed when the buffer size is between 0.5 BDP and approximately 1–2 BDP. For buffer sizes outside this range, the dominance of the flow with the shorter RTT becomes more pronounced.

In contrast, RTT fairness for BBR shows much greater dependence on the buffer size than on the RTT ratio. For deep buffers, the flow with the longer RTT consistently dominates, capturing up to 90% of the available bandwidth. This is also a common conclusion in most papers that evaluate BBR performance. However, this dominance becomes less pronounced in shallow buffer scenarios. For buffer sizes between 0.05 and 0.5 BDP, BBR demonstrates more equitable bandwidth sharing, particularly when the RTT ratio is moderate. Notably, even when the RTT of the second flow increases to 200 ms, the shorter flow still obtains approximately 30% of the total bandwidth, indicating a reduction in the degree of unfairness compared to deep buffer configurations.

The results show significant differences between scenarios where the RTT ratio is the same but the ratio of buffer size to the RTT of the longer flow varies. The bandwidth-delay product (BDP) is always calculated based on the 20 ms RTT flow, meaning that 1 BDP corresponds to buffering up to 20 ms worth of data. In the scenario where flows with 2 ms and 20 ms RTTs compete, the dominance of the longer RTT flow is more pronounced and occurs at much smaller buffer sizes compared to the scenario with flows of 20 ms and 200 ms RTTs. This is because buffer occupancy increases the measured RTT interval observed by the BBR algorithm, resulting in an inflated delivery rate estimate and, consequently, an inaccurate bandwidth estimation. When the second flow has a 2 ms RTT, the buffer appears relatively deep for that flow, causing a significant delay overhead that amplifies the dominance of the long-RTT flow even at shallow buffer sizes. This also explains why buffers sized between 2 and 5 BDP provide RTT fairness when the longer RTT flow approaches 200 ms.

Absolute RTT strongly affects buffer perception. A fixed queue of size *B* bytes corresponds to B/(C·RTT) BDP for a flow with round-trip time RTT over a link of capacity *C*. Thus, the same buffer appears deep to short-RTT flows but shallow to long-RTT flows. For example, at 1 Gbps, a 2 MB buffer equals 8 BDsP for a 2 ms flow but only 0.08 BDP for a 200 ms flow. The short-RTT flow, having a small BDP, perceives the buffer as large and can easily fill it. In contrast, the long-RTT flow has a much larger BDP and perceives the same buffer as insufficiently small, leading to frequent packet loss and an inability to sustain its sending rate.

**Figure 5:**
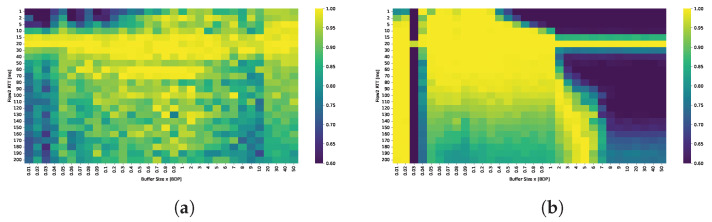
Fairness (Jain’s index) of (**a**) TCP CUBIC and (**b**) TCP BBR under varying buffer sizes and RTTs.

The comparison of the Jain’s index maps presented in Figure 5 confirms that TCP CUBIC achieves fairness only when the RTT ratio is small. The best results in terms of RTT fairness are observed for buffer sizes in the range of 0.8 to 2 BDP. Conversely, BBR can maintain fairness for shallow buffers, provided that the buffers remain relatively shallow across all connections.

#### 4.1.3. Retransmission Ratio

**Figure 6:**
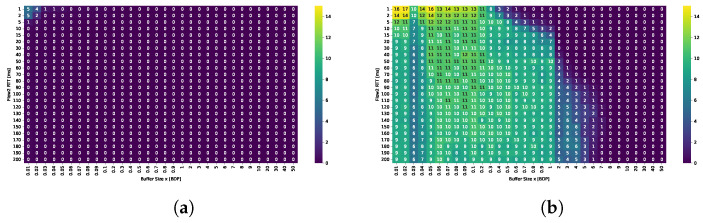
Retransmission ratio [%] of (**a**) TCP CUBIC and (**b**) TCP BBR under varying buffer sizes and RTTs.

The primary drawback of BBR when operating with shallow buffers is evident in the average retransmission ratio (see Figure 6). For TCP Cubic, the retransmission rate remains negligible, not exceeding 0.5%. In contrast, BBR exhibits a retransmission ratio of approximately 10% when the buffer size is below 2 BDP. As BBR does not actively monitor packet losses, retransmissions are triggered only upon timeout. Nevertheless, it should be noted that BBR effectively utilizes even small buffers, delivering high throughput and maintaining RTT fairness. As a result, the goodput achieved with BBR is expected to exceed that of Cubic in those scenarios.

#### 4.1.4. Queue Occupancy and RTT Behavior

**Figure 7:**
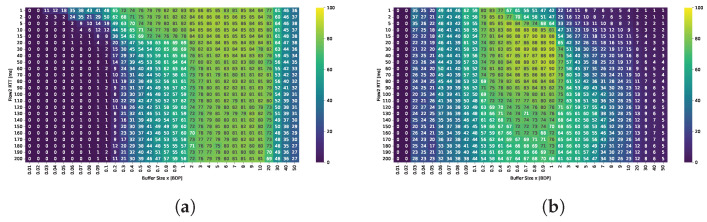
Average queue occupancy [%] under (**a**) TCP CUBIC and (**b**) TCP BBR over varying buffer sizes and RTTs.

The analysis of the average queue occupancy confirms the findings reported earlier. Poor bandwidth utilization in TCP Cubic is mainly due to insufficient buffer capacity. Cubic is unable to ramp up its sending rate when the buffer size is smaller than 0.3 BDP, which corresponds to approximately 750 KB, or about 500 packets assuming a packet size of 1500 bytes, see Figure 7. At this threshold, the queue becomes highly unstable, fluctuating rapidly between empty and full. As the buffer size increases, the queue becomes steadier. However, the larger the RTT differences between competing flows, the oscillations in queue length are more pronounced, requiring even larger buffers to achieve stability. In general, deeper buffers lead to more consistent queue behavior, regardless of RTT disparity. However, this comes at the cost of increased queuing delay, which can exceed the base RTT by more than an order of magnitude. For Cubic, increasing buffer sizes beyond 20 BDP does not yield further improvements, as the algorithm is unable to fully utilize such deep buffers.

BBR utilizes the majority of the buffer only when its size is below 1 BDP. For larger buffers, average utilization typically reaches up to approximately 1.5 BDP. The presence of long-RTT flows tends to increase buffer occupancy; however, in most cases, the average queue size does not exceed 2 BDP. Since BBR is insensitive to packet loss, larger buffers prevent excessive loss rates; however, this comes at the cost of degraded RTT fairness, as longer buffers exacerbate the dominance of flows with higher round-trip times.

The RTT observed by each flow is closely correlated with the average queue occupancy, and both flows are similarly affected. For Cubic, the increased RTT primarily contributes to additional latency experienced at the receiver. In contrast, for BBR, accurate RTT measurement is critical to the algorithm’s operation.

A detailed analysis of RTT measurements across all connections shows that capturing the minimum RTT, close to the baseline link delay, is generally not problematic; in most scenarios, it is accurately recorded. However, the main challenge arises during the ProbeBW phase, where the current RTT is often inflated because of bufferbloat. This inflated RTT significantly affects the accuracy of the bandwidth estimate. Although all flows experience additional delays caused by queueing, the relative impact varies depending on the RTT of the flow path. Consequently, the inflated delay constitutes a larger proportion of the total RTT for shorter-delay flows, leading to greater distortion in bandwidth estimation.

#### 4.1.5. Detailed Analysis of Shallow and Deep Buffer Scenarios

To provide a detailed explanation of how buffer size influences BBR’s behavior in terms of fairness, we present an in-depth analysis of two connections with RTTs of 20 ms and 60 ms sharing a common link. The results show that when the buffer size is set to 0.3 BDP, bandwidth is shared equally between the two flows. However, as the buffer size increases to 8 BDP, the longer RTT flow begins to dominate the bandwidth, indicating a clear shift in fairness dynamics with buffer depth.

**Figure 8:**
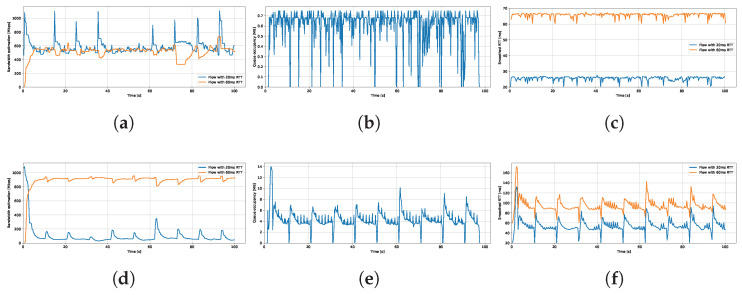
TCP BBR dynamics under shallow = 0.3 BDP (top row) and deep 8 BDP (bottom row) buffer scenarios: (**a**,**d**) bandwidth estimation [Mbps], (**b**,**e**) queue occupancy [MB], (**c**,**f**) RTT observed by both flows.

TCP BBR behavior over time under different buffer sizes is presented in Figure 8. The top row shows bandwidth estimation, queue occupancy, and measured RTT in a shallow buffer scenario (0.3 BDP). The bottom row presents the same metrics for a deep buffer scenario (8 BDP).

When the queue can buffer only up to 6 ms of traffic (0.3 BDP), the measured RTT remains close to the minimum (i.e., the actual path delay). As a result, the delivery rate measurements accurately reflect the true bandwidth capacity, allowing the bandwidth estimation to converge to the available rate. Although differences in RTT influence the speed at which each flow updates its estimate, they do not affect the accuracy of the final estimation.

While the ProbeRTT phases tend to synchronize across both flows, the shorter RTT flow is more likely to enter and exit this phase slightly earlier. For the shallow buffer scenario, the prominent peaks observed in the figure result from the 60 ms RTT flow remaining in the ProbeRTT phase slightly longer, thereby allowing the 20 ms flow to probe for and utilize additional bandwidth temporarily. Due to the shallow queue, any reduction in sending rate by both flows immediately reveals the full link capacity, allowing the shorter flows to gain a larger share of the bandwidth. Nevertheless, the 20 ms RTT flow rapidly converges to its correct bandwidth share.

A larger buffer can absorb significantly more inflight data without inducing packet loss; however, it introduces additional queuing delay. For a buffer size of 8 BDP, the average queue occupancy oscillates around 5 MB (2 BDP), resulting in approximately 30–40 ms of additional queueing delay for both flows. Under these conditions, the long RTT flow sustains a higher bandwidth estimate and continues transmitting at an elevated rate. In contrast, the short RTT flow experiences an increased RTT due to queuing, but its bandwidth estimate remains relatively low, leading to persistent unfairness.

In addition to receiving acknowledgments less frequently, long RTT flows may experience further inflation of the delivered data metric due to delayed ACKs. The extended feedback interval not only slows the rate at which the bandwidth estimate is updated, but also allows the flow to remain in the ProbeBW phase longer, leading to further queue buildup. As acknowledgments continue to arrive, BBR interprets the sustained delivery as an indication that the elevated sending rate is appropriate. This results in an overestimation of the available bandwidth, as observed for deep buffer scenario. Due to the slower feedback loop associated with longer RTTs, the overestimation persists and the effect compounds over time.

Shallow buffers promote faster feedback, constraining both flows to compete within a limited queueing space and closer to the path RTT. This pressure helps to reset bandwidth overestimations more quickly. Additionally, shallow queues enforce similarly brief STARTUP phases for both flows. When the buffer is deep, the long RTT flow remains in the STARTUP phase significantly longer, leading to higher peaks in queue occupancy and greater delay.

#### 4.1.6. Summary

In the two-flow experiment, we evaluated how buffer size and RTT asymmetry influence bandwidth utilization and fairness for TCP Cubic and TCP BBR. The results show that TCP Cubic requires a buffer size of at least 0.3 BDP to achieve high bandwidth utilization, while BBR maintains high utilization regardless of buffer size or RTT ratio. Cubic exhibits RTT unfairness when competing flows have dissimilar RTTs; shorter RTT flows consistently dominate, and fairness only improves when the RTT ratio is small. For Cubic, optimal performance in terms of bandwidth utilization, queue stability and fairness is observed with buffer sizes between 0.8 and 2 BDP, and degrades sharply below 0.5 BDP.

In contrast, BBR’s RTT fairness is more sensitive to buffer sizing than to RTT differences. Fair bandwidth sharing is achieved only when buffer sizes are sufficiently shallow, typically below 0.5 BDP per flow, to prevent the inflight data of each flow from exceeding its congestion window gain. Under these conditions, BBR successfully balances delivery rates and avoids long RTT flow dominance, but at the expense of higher retransmission rates and greater buffer occupancy. Unlike Cubic, which tends to fill the available buffer to maximize throughput, BBR deliberately limits inflight data, rarely exceeding 2 BDP regardless of buffer capacity.

### 4.2. Modeling RTT Fairness: Multi-Flow Scenario

In this scenario, we further investigated how sender distribution, buffer sizing, and RTT heterogeneity influenced transmission performance. The goal was to assess whether the number of flows with longer or shorter RTTs affected fairness—specifically, whether BBR’s fairness continued to depend primarily on buffer size, and Cubic’s on the RTT ratio, under varying numbers of shorter and longer RTT flows.

A dumbbell topology was used in which 10 senders communicated with 10 corresponding receivers over a shared bottleneck link with a fixed one-way propagation delay of 2 ms. The senders were divided into two groups: Group 1 maintained a constant round-trip time (RTT) of 20 ms, while Group 2 was assigned variable RTTs ranging from 2 to 200 ms. The RTT ratio between the two groups varied symmetrically from 10:1 to 1:1 and back to 1:10. The proportion of senders from Group 1 ($n_{1}$) varied from 0% to 100%, with the remaining senders assigned to Group 2 ($n_{2}$). Each flow transmitted long-lived TCP traffic to maintain a steady congestion state throughout the experiment.

For all simulations, per-flow metrics were collected, including average throughput, retransmission ratio, and mean latency, along with aggregated system-wide metrics. All values were averaged across multiple runs to ensure result stability and reduce noise. Inter-group fairness was assessed using the Symmetric Throughput Difference Ratio (STDR), which compared the average throughputs of Group 1 and Group 2. Intra-group and overall fairness were evaluated using Jain’s fairness index.

To quantify how much bandwidth each group received relative to its fair share, the Relative Throughput Share (RTS) was calculated for each group as:

(4) $$RTS=\frac{\sum thr_{g}}{n_{g}\cdot thr}\times 100\%,$$

where $\sum thr_{g}$ was the total throughput of group *g*, $n_{g}$ was the number of flows in group *g*, and thr was the average throughput across all flows. Values above 100% indicate that the group receives more than its proportional share of bandwidth.

#### 4.2.1. Bandwidth Utilization

Preliminary analysis showed that the buffer size had the greatest impact on performance and achieved fairness. Therefore, the plot analysis was structured according to buffer size categories to better illustrate these effects. Buffers smaller than 0.5 BDP were categorized as small, those ranging from 0.5 to 2 BDP as medium, and those larger than 2 BDP as large.

**Figure 9:**
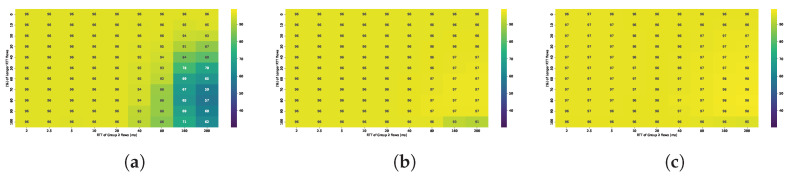
Average per-flow throughput [Mbps] for TCP Cubic under (**a**) small, (**b**) medium, and (**c**) large buffer sizes.

Figure 9 shows the average throughput per TCP Cubic flow (in Mbps), which closely reassembles the average bandwidth utilization given the presence of 10 active flows and a 1 Gbps link. The results are presented for small (0.1 BDP), medium (1 BDP), and large (5 BDP) buffer sizes.

In the previous scenario, Cubic achieved high bandwidth utilization only when the relative buffer size was at least 0.3 BDP with respect to any of the active flows. In the multi-flow setting, a buffer size of 0.05 BDP proved insufficient, whereas a buffer of 0.1 BDP generally ensured acceptable performance. However, in scenarios dominated by long-RTT flows, utilization could still drop to 60–70%. The results indicated that as the number of concurrent streams increased, the buffer size requirements decreased. For example, while two flows with RTT = 20 ms were unable to utilize more than 80% of the bandwidth with a 0.1 BDP buffer, ten such flows were able to fully saturate the link. In contrast, when the RTT of the concurrent flows increased, a greater number of shorter RTT flows was required to fully utilize the available bandwidth.

A buffer of 0.5 BDP was sufficient to maintain high utilization in all tested configurations; however, in scenarios where all flows had RTTs of 160 ms or 200 ms, utilization fell below 90%. Increasing the buffer to 1 BDP, thus, providing at least 0.1 relative BDP for the longest flows, yielded a modest improvement in these long-path scenarios, while further increases did not result in significant gains.

These results suggest that, to ensure high bandwidth utilization, the buffer size available to each active stream must be at least 10% of its BDP. When this condition is not met for all flows, overall utilization depends on two factors: the number of flows that do satisfy the condition, and the RTT of those that do not. In such cases, the longer the RTTs of under-buffered flows, the more short-RTT flows are required to compensate and maintain high link utilization.

TCP BBR maintains high bandwidth utilization—exceeding 90%, even with very small buffers (as low as 0.05 BDP) across all tested scenarios. While increasing the buffer size does not significantly impact overall utilization, it substantially alters how bandwidth is distributed between shorter- and longer-RTT flows.

#### 4.2.2. Throughput and Fairness

##### Intra-Group Fairness

To better understand the origins of RTT-induced unfairness, we first examine how well fairness is maintained among flows sharing the same RTT (intra-group fairness).

Both Cubic and BBR handle fairness reasonably well among flows with identical RTTs. However, Cubic shows slightly greater difficulty in maintaining fairness within groups of flows sharing the same RTT (intra-group fairness). Analysis of Jain’s index, calculated separately for Group 1 and Group 2, indicates that fairness is primarily affected by buffer size, with performance deteriorating as the buffer increases. Additionally, a larger number of flows within a group further complicates a fair bandwidth distribution. While the longest RTT values also influence fairness, this effect becomes significant only in deep buffer scenarios, where the dominating shorter-RTT flows experience a decline in intra-group fairness.

For BBR, high fairness within both Group 1 and Group 2 flows is maintained across the vast majority of scenarios. Even in cases where overall fairness appears degraded, the imbalance primarily results from inter-group disparities rather than intra-group unfairness. This is supported by Jain’s index values calculated separately for each group, which exceed 0.95 in at least 95% of cases, indicating negligible variation within groups. The lowest fairness levels were observed only when the buffer size exceeded 1 BDP. A slight decline in fairness was also associated with scenarios where one group dominated in size while the opposing group consisted of flows with significantly longer RTTs.

##### TCP Cubic

Table 1 presents the average values of key performance metrics for TCP Cubic, including overall fairness (Jain’s Index), intra-group fairness (Jain’s Index), average throughput, and the average throughput of shorter- and longer-RTT flow groups, across different buffer size categories. These results allow for direct comparison of how buffer depth impacts both fairness and bandwidth allocation among flows with heterogeneous RTTs.

An analysis of the average Jain’s index reveals that Cubic performs worst in terms of fairness when buffer sizes are very small. In these cases, shorter RTT flows can consume up to 150% of the ideal share of the bandwidth. This behavior stems from Cubic’s design: flows with shorter RTTs experience faster congestion window growth, enabling them to fill the buffer more quickly. Because small buffers cannot accommodate many packets from longer-RTT flows, the queue becomes dominated by shorter-RTT traffic, further limiting access to bandwidth for longer-RTT flows. As a result, longer-RTT flows not only fail to utilize their fair share of the bandwidth, but a significant portion is additionally captured by shorter flows.

For buffer sizes in the range of 0.5 to 2 BDP, Cubic demonstrates the highest level of fairness, as indicated by both the overall Jain’s index and the reduced disparity in average throughput between shorter- and longer-RTT flows. Although shorter-RTT flows still exhibit a throughput advantage—on average approximately 20% higher than the overall mean—the degree of imbalance is substantially lower compared to smaller buffer scenarios. Notably, the absolute throughput of shorter flows remains comparable to that observed with very small buffers, whereas longer-RTT flows benefit significantly from the increased buffer capacity, achieving nearly twice the throughput relative to small buffer configurations.

As the buffer size increases, the advantage gained by shorter-RTT flows becomes more pronounced. In these scenarios, bandwidth utilization approaches 100%; however, due to the faster growth of the short flows’ congestion window, they tend to occupy a larger portion of the buffer, thus, ensuring a greater share of available bandwidth. Since the only limiting mechanism in Cubic is packet loss—which, in the absence of AQM, occurs only upon buffer overflow—this imbalance intensifies with increasing buffer size.

**Figure 10:**
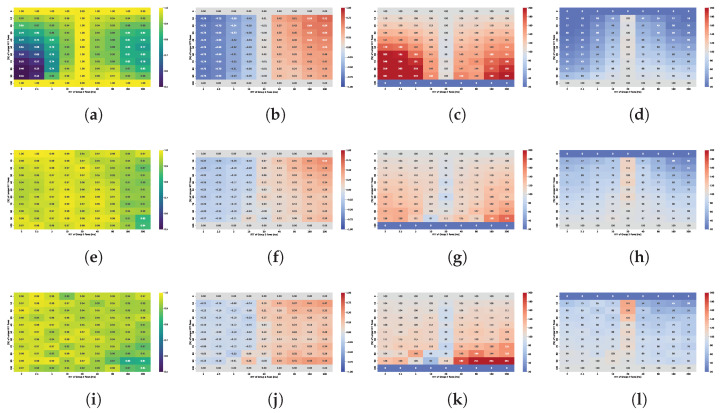
Comparison of overall fairness (Jain’s Index)—(**a**,**e**,**i**), STDR—(**b**,**f**,**j**), Relative Throughput Share (RTS) for shorter-RTT flows—(**c**,**g**,**k**), and Relative Throughput Share (RTS) for longer-RTT flows—(**d**,**h**,**l**) across three buffer categories: small (**a**–**d**), medium (**e**–**h**), and large (**i**–**l**) for TCP Cubic.

Figure 10 presents heatmaps showing: (i) overall fairness (Jain’s Index) in subfigures (a,e,i); (ii) inter-group fairness (STDR) in (b,f,j); (iii) Relative Throughput Share (RTS) for the group of flows with shorter RTT in each scenario, shown in (c,g,k); (iv) Relative Throughput Share (RTS) for the group of flows with longer RTT in each scenario, shown in (d,h,l). These metrics capture complementary aspects of fairness and performance across varying RTT and sender group configurations.

The plot analysis and analysis of averaged fairness confirms that the highest fairness and more balanced bandwidth distribution between groups occur when the buffer size is between 1–2 BDP. While shorter RTT flows generally dominate, their advantage is less pronounced within this buffer range compared to other configurations.

For shallow buffers, overall fairness deteriorates significantly in the presence of RTT heterogeneity. Specifically, Jain’s fairness index decreases (i) as the ratio between longer- and shorter-RTTs increases, and (ii) when the numbers of shorter- and longer-RTT flows are approximately equal. Cubic generally preserves fairness among flows with identical RTTs, so the observed disparities primarily result from interactions between flows with differing RTTs.

For a buffer size of 0.1 BDP, high fairness values (close to one) are maintained when all flows share the same RTT. A symmetry can be observed across the plots, where the fairness index remains similar for mirrored RTT ratios, regardless of the absolute RTT values. This suggests that the RTT ratio is the dominant factor influencing fairness under these conditions. For a fixed RTT ratio, overall fairness is lowest when shorter- and longer-RTT flows are evenly distributed and improves as the distribution becomes more imbalanced in either direction. An additional effect is also observed—the disproportionate advantage gained by a small number of short RTT flows, but only when the relative buffer is medium sized for those flows.

In shallow buffer environments, shorter-RTT flows tend to dominate the queue, allowing them to significantly exceed their fair share. In extreme cases, a single short RTT flow can monopolize nearly half of the total available bandwidth, while long RTT flows may be limited to just 60% of their expected share.

The Symmetric Throughput Difference Ratio (STDR) highlights the delay-induced asymmetry between Group 1 and Group 2 flows. On the left side of the plot, Group 1 is a group of longer RTT flows, while on the right side, it represents shorter-RTT flows—hence the change in sign, despite similar absolute values. The magnitude of this asymmetry primarily depends on the RTT ratio: the greater the ratio, the more pronounced the difference. The disparity also tends to increase with the number of shorter-RTT flows. However, as with overall fairness, in the presence of extremely short RTT connections, the number of such flows appears to have little additional effect, their presence alone is sufficient to introduce a comparable level of asymmetry across scenarios.

The Relative Throughput Share (RTS) for shorter-RTT flows increases with both the RTT ratio (longer to shorter flows) and the decreasing number of shorter flows. Similarly, from the perspective of the dominated group, i.e., longer-RTT flows, the RTS increases as the number of longer-RTT flows grows. However, both values are strongly influenced by the RTT ratio: shorter flows gain more as the ratio increases, while longer flows benefit less under high RTT disparity. Although isolated shorter-RTT flows can obtain a disproportionately large share of the bandwidth, a larger number of longer-RTT flows can collectively improve their total bandwidth share. These findings indicate that flow composition and group distribution must be carefully considered when evaluating fairness in shallow-buffer regimes.

For buffer sizes of 1 and 2 BDP, overall fairness remains very high across all scenarios. It degrades slightly in the presence of longest RTT flows; however, neither the RTT ratio nor the sender group distribution significantly influences the results. In contrast, a dependence on RTT is still observed for the 0.5 BDP buffer, indicating that this size is insufficient for Cubic to ensure consistent fairness.

The STDR values are noticeably lower than in small buffer scenarios, suggesting a reduced level of asymmetry. This metric is primarily influenced by the RTT ratio and, as in previous cases, increases with the number of shorter-RTT flows. In scenarios involving the longest RTTs, asymmetry becomes more pronounced, likely due to the buffer still being relatively small in comparison to the BDP of long RTT flows, which hinders their ability to achieve higher throughput.

The imbalance in RTS follows a pattern similar to that observed in small-buffer scenarios, although the gains of dominating flows are significantly lower. A single longer-RTT flow can still obtain at least 50% of its ideal share, while nine such flows can achieve over 95% each.

As buffer size increases, overall fairness declines. While a 5 BDP buffer still maintains a relatively high Jain’s index of around 0.95, this value drops below 0.8 on average when the buffer size reaches 20 BDP. In contrast to small buffer scenarios, where fairness is clearly influenced by the RTT ratio, no consistent trend with respect to RTT ratio is observed in large-buffer configurations.

Larger buffers also amplify the STDR, especially in scenarios where Group 2 flows have RTTs exceeding 20 ms (i.e., the right-hand side of the heatmaps). Although STDR patterns remain broadly similar to those observed for medium buffers, the influence of RTT ratio diminishes as buffer size increases. Instead, the most significant throughput disparities emerge in scenarios with highly unbalanced group compositions, particularly when the difference between the number of short and long RTT flows is greatest.

A similar trend is observed in high-RTT scenarios, where the RTS is no longer strongly driven by RTT ratio but rather by the flow group distribution. As in shallow buffer configurations, a small number of shorter-RTT flows consistently capture a disproportionately large share of the bandwidth, and this effect becomes even more pronounced with larger buffers. From buffer sizes of 10 BDP onward, the additional bandwidth gained by shorter-RTT flows increases as their number decreases. This trend is especially evident in scenarios involving the longest RTT connections. These results clearly indicate that large buffers negatively impact fairness, especially for long RTT flows, and this effect persists regardless of traffic composition or network conditions.

##### TCP BBR

In the case of BBR, an analysis of the average values across the three buffer categories clearly shows that fairness decreases as the buffer size increases (see Table 2).

For small buffers, Jain’s index is highest—approaching one—and the average throughputs achieved by shorter- and longer-RTT flows are nearly identical. With medium buffers, the fairness metric drops significantly and the average throughput of shorter flows is nearly three times lower than that of longer ones. For large buffers, this difference increases to a factor of ten, and the overall Jain’s index falls to approximately 0.6. These results clearly indicate that the fairness between the BBR flows is severely compromised in medium and large buffer configurations.

**Figure 11:**
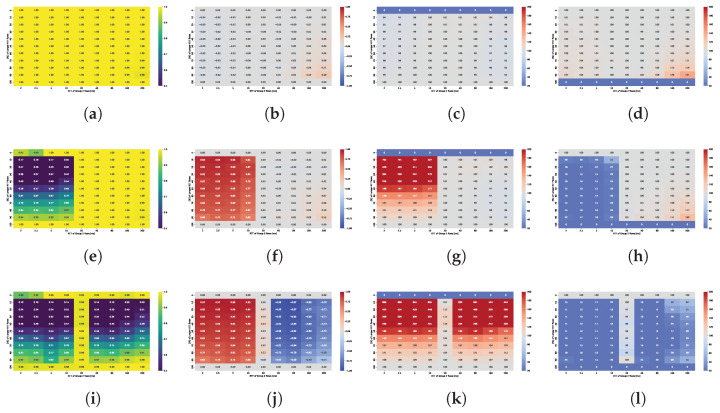
Comparison of overall fairness (Jain’s Index)—(**a**,**e**,**i**), STDR—(**b**,**f**,**j**), Relative Throughput Share (RTS) for longer-RTT flows—(**c**,**g**,**k**), and Relative Throughput Share (RTS) for shorter-RTT flows—(**d**,**h**,**l**) across three buffer categories: small (**a**–**d**), medium (**e**–**h**), and large (**i**–**l**) for TCP BBR.

Figure 11 presents heatmaps showing: (i) overall fairness (Jain’s Index) in subfigures (a,e,i); (ii) inter-group fairness (STDR) in (b,f,j); (iii) Relative Throughput Share (RTS) for the group of flows with longer RTT in each scenario, shown in (c,g,k); and (iv) Relative Throughput Share (RTS) for the group of flows with shorter RTT in each scenario, shown in (d,h,l).

TCP BBR achieves near-perfect overall fairness in small buffer scenarios, with Jain’s index values consistently close to one. Only slight deviations are observed in configurations that involve the longest RTT flows. Similarly, the Symmetric Throughput Difference Ratio (STDR) remains largely stable, indicating minimal imbalance. Bandwidth distribution analysis reveals that in scenarios involving the longest and shortest RTT flows, **shorter-RTT** flows gain a modest bandwidth advantage, which becomes more pronounced as their number decreases. A single 20 ms flow can achieve up to 140% of its ideal bandwidth share when competing against nine 200 ms flows.

Analysis of medium buffer scenarios (0.5–2 BDP) revealed a consistent degradation in overall fairness beginning at a buffer size of approximately 0.5 BDP. At this threshold, degradation was most pronounced in configurations involving the shortest RTT flows (e.g., 2 to 2.5 ms), for which the absolute buffer size corresponds to a relatively large share of their BDP—up to 5 BDP in relative terms. As the buffer size increased, fairness degradation also extended to scenarios involving higher RTTs as well.

A key factor contributing to fairness deterioration was the presence of a single, longer-RTT flow. This flow introduced the greatest throughput disparity, often achieving nearly 80% of the available bandwidth. As the number of longer-RTT flows increased, overall fairness, as measured by Jain’s index, also improved. This trend reflected BBR’s ability to maintain fairness among flows with similar RTT (intra-group fairness). However, analysis of the STDR showed that fairness between the RTT groups (inter-group fairness) remained poor. Even as the number of longer RTT flows increased, significant throughput disparities between groups persisted.

This was further evidenced by the Relative Throughput Share (RTS). For example, with a buffer size of 1 BDP and a 9:1 ratio of shorter- to longer-RTT flows, each shorter-RTT flow achieved approximately 26% of its ideal bandwidth share. However, when only five shorter-RTT flows were active (i.e., a 50/50 distribution), each received just over 10% of their expected share. Some improvement was observed as the number of longer-RTT flows increased, with RTS for the single, shorter-RTT flow rising to 22% when competing against nine longer-RTT flows.

Interestingly, neither fairness metric was found to depend on the RTT ratio itself, but rather on the buffer size relative to the RTT of the active flows. Once the buffer became large enough that BBR flows were constrained by the congestion window (i.e., when the inflight data exceeded 2 × estimated BDP), bandwidth imbalance was increasingly dominated by longer-RTT flows. In these configurations, a substantial drop in retransmission ratios was observed for the congestion window–limited group, confirming that BBR was no longer transmitting without constraint.

The RTT ratio did not influence the fairness between groups as well. STDR values remained similar across scenarios with varying RTT ratios but consistently decreased as the number of longer-RTT flows increased. Likewise, the RTS of longer-RTT flows declined with increasing group size. However, when the RTS was analyzed from the perspective of shorter-RTT flows, a nonlinear dependency on group composition emerged. The lowest throughput share occurred in balanced scenarios (i.e., 50/50 distribution), while configurations with a greater imbalance—whether consisting of more shorter or longer RTT flows—led to improved bandwidth shares for the shorter RTT group.

For deep buffer scenarios, the described patterns extend across all configurations, regardless of the RTT ratio. Overall fairness is lowest when only one shorter-RTT flow is present and improves as the number of longer-RTT flows increases—mainly due to BBR’s high intra-group fairness. The STDR follows a similar trend, depending exclusively on the sender distribution. Comparable conclusions apply to bandwidth imbalance, which is likewise driven primarily by the distribution of flows with differing RTTs.

### 4.2.3. Retransmission Ratio

The retransmission ratio for TCP Cubic is negligible. It only reaches up to 2% for extremely small buffers (0.05 BDP) and short flows (e.g., 2 ms RTT); in other cases, it remains close to zero. While shorter buffers slightly increase the retransmission ratio, it rarely exceeds 0.2%.

**Figure 12:**
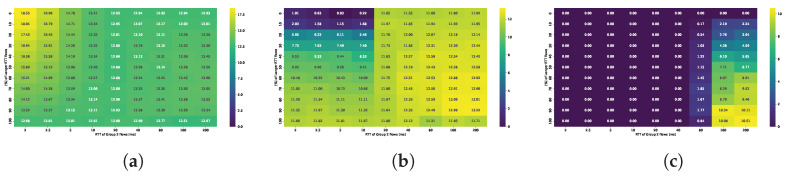
Average retransmission ratio [Mbps] for TCP BBR under (**a**) small, (**b**) medium, and (**c**) large buffer sizes.

TCP BBR exhibits significantly higher retransmission ratios. Figure 12 presents the overall retransmission ratio for small, medium and large buffers.

For the smallest buffers, the ratio of retransmitted packets reaches up to 20%, although in most scenarios it remains below 15%. This behavior stems from BBR’s design, which does not incorporate loss detection; as a result, it continues transmitting data even when buffer capacity is exceeded. A breakdown by flow group reveals that retransmission ratios are typically a few percentage points higher for shorter RTT flows. Notably, this effect does not appear to depend on the number of such flows; however, the overall retransmission level increases as the proportion of short RTT flows rises.

The retransmission ratio decreases substantially as the buffer size increases. It is important to note that the elevated retransmission rate observed with BBR under small buffer conditions does not imply lower goodput. On the contrary, BBR achieves better bandwidth utilization in these scenarios. According to [26], its goodput remains higher than that of Cubic despite the increased retransmissions.

### 4.2.4. Queue Occupancy and RTT Behavior

**Figure 13:**
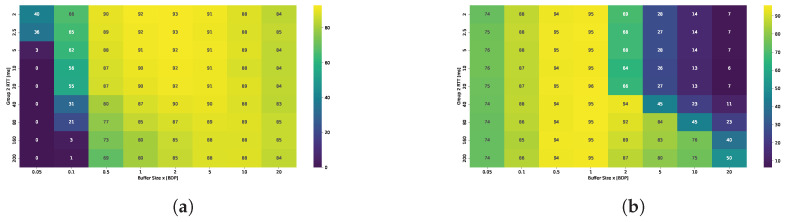
The average queue occupancy [%] for (**a**) TCP Cubic, (**b**) TCP BBR.

The sender distribution does not significantly impact the observed queue utilization, so similar conclusions can be drawn as in the previous experiment. Figure 13 shows the average queue occupancy in a 50/50 sender distribution scenario for both congestion control algorithms.

Cubic struggles to fully utilize the available bandwidth when buffers are very small, leading to lower buffer occupancy. However, as the number of shorter RTT flows—for which the relative buffer size is effectively larger—increases, bandwidth utilization improves, resulting in higher average queue occupancy. Cubic tends to completely fill the available buffer, leading to high utilization in most configurations. Consequently, average queue sizes often reach the buffer limit.

BBR, in contrast, fully utilizes only small buffers. Utilization of larger buffers beyond 2 BDP depends on both the number of long RTT flows and the RTT values of the active flows. Across scenarios with varying sender distributions, the queue occupancy pattern remains similar to that shown in Figure 13, though the presence of more long RTT flows leads to a slight increase in queue occupancy.

The average RTT observed by each flow in both algorithms is closely correlated with queue occupancy. The additional delay, i.e., the difference between observed RTT and path RTT, is consistent across all streams and primarily determined by buffer occupancy. No significant differences were observed with respect to group composition or RTT ratio. All active flows experience similar additional latency, with the mean RTT increasing proportionally to average queue occupancy.

Unfortunately, when the buffer is fully utilized, it introduces a similar absolute latency to both long and short RTT flows but has a disproportionately greater effect on the latter. A buffer sized at 1 BDP, calculated for a 20 ms RTT, introduces approximately 20 ms of queuing delay. While this adds only 10% to the delay of flows with a baseline RTT of 200 ms, it increases the total RTT tenfold for flows with a baseline of 2 ms. Although such added delay does not directly affect the behavior of the Cubic algorithm, it can significantly degrade the quality of experience, especially for interactive or real-time applications. Larger buffers also increase the likelihood that BBR fails to accurately register the minimum RTT, further degrading the performance of short-RTT flows. This negatively affects the proper BBR’s estimation of bandwidth and reinforces the dominance of long-RTT flows.

### 4.2.5. Summary

In the multi-flow experiment, we examined how buffer size, RTT ratio, and sender group composition affect bandwidth utilization and fairness in TCP Cubic and BBR. TCP Cubic requires a buffer size of at least 0.1 BDP to utilize bandwidth effectively, with the best results in terms of throughput, overall fairness, and RTT fairness achieved at 1 to 2 BDP. Under small and medium buffer conditions, fairness was found to be strongly influenced by the RTT ratio, with flows experiencing shorter RTTs dominating under asymmetric conditions. Furthermore, fairness was observed to reach its lowest point at a 50/50 sender group distribution, suggesting a concave relationship between group balance and fairness.

By contrast, BBR achieves high link utilization even with minimal buffering, consistently delivering high throughput across all RTTs. Moreover, only the smallest buffers allow for RTT fairness provisioning. Although BBR tends to exhibit a higher retransmission ratio, especially under shallow buffers, its goodput remains comparable to that of Cubic due to its high link utilization. Unlike Cubic, BBR’s fairness is not directly governed by the RTT ratio, but rather by the interaction between RTT and buffer size. When flows are capped by their congestion window gain, longer RTT flows receive disproportionately more bandwidth. In such cases, sender group distribution becomes the dominant factor in determining how throughput is shared.

**Figure 14:**
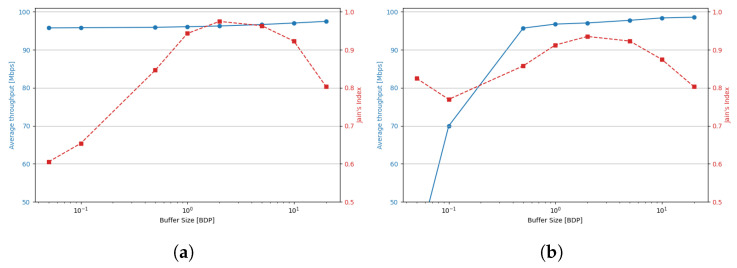
Comparison of average throughput and overall Jain’s index for (**a**) Group2 RTT = 2 ms and (**b**) Group2 RTT = 200 ms under varying buffer sizes and 50/50 sender distribution (TCP Cubic).

**Figure 15:**
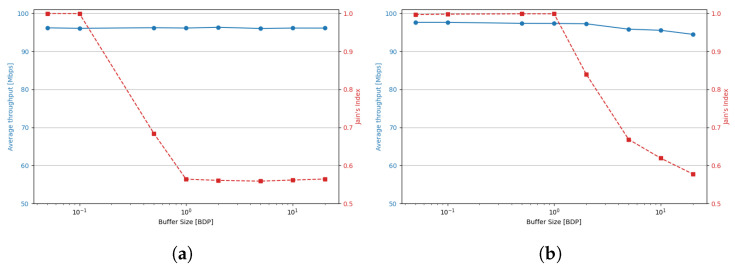
Comparison of average throughput and overall Jain’s index for (**a**) Group2 RTT = 2 ms and (**b**) Group2 RTT = 200 ms under varying buffer sizes and 50/50 sender distribution (TCP BBR).

Figure 14 and Figure 15 illustrate the dependence of average throughput and Jain’s fairness index on buffer size for two RTT configurations (2 and 200 ms) under a balanced (50/50) sender distribution. The results confirm that fairness improvements are not achieved at the cost of throughput: average per-flow throughput remains nearly constant at around 100 Mbps in most scenarios. Only Cubic shows a notable throughput drop at very small buffers (<0.5 BDP). In contrast, Jain’s index reveals distinct fairness peaks: for BBR, the highest fairness is observed with shallow buffers (<0.5 BDP), while Cubic achieves its best fairness with moderate buffers (1–2 BDP). These findings confirm that careful buffer sizing can improve RTT fairness while maintaining high utilization, highlighting practical operating regions for each algorithm.

Table 3 summarizes the best operating conditions for achieving fairness in TCP Cubic and TCP BBR, providing a concise comparison of how buffer size, RTT ratio, and sender distribution shape fairness outcomes.

## 5. Conclusions

This study investigated the impact of buffer sizing and RTT heterogeneity on fairness and bandwidth allocation in TCP congestion control, focusing on BBR and Cubic algorithms. Our results demonstrate that buffer size plays a critical role in shaping both overall fairness and intra-/inter-group bandwidth distribution among flows with diverse RTTs.

Our results confirm prior observations that TCP Cubic performs best with medium to large buffers, whereas TCP BBR achieves optimal utilization with small buffers. However, our findings challenge the common belief that RTT ratio is the primary determinant of RTT unfairness in BBR. We also expand upon the established claim that for TCP Cubic, with small buffers, the number of short-RTT flows has the greatest impact on performance, whereas with large buffers, variations in the number of long-RTT flows become more influential.

Both protocols generally preserve intra-group fairness, suggesting that fairness degradation primarily stems from RTT heterogeneity across groups. For TCP Cubic, providing a medium-sized buffer of 1–2 BDP is key to achieving both high overall fairness and RTT fairness. Although this buffer size does not eliminate the bandwidth advantage of shorter-RTT flows, it significantly improves performance across fairness metrics. In this buffer range, overall fairness remains high and relatively insensitive to RTT ratio, though RTT still influences both the throughput difference between shorter- and longer-RTT flows and their Relative Throughput Shares (RTS), especially as the number of longer-RTT flows increases.

In small buffer scenarios, Cubic’s dependence on RTT ratio and sender group composition becomes more pronounced. Overall fairness degrades with increasing RTT ratio and shows a nonlinear dependency on sender distribution, reaching its lowest point when shorter- and longer-RTT flows are evenly balanced. The Symmetrical Throughput Difference Ratio (STDR) and Relative Throughput Share (RTS) exhibit similar trends as in medium buffer cases, but the magnitude of imbalance is more severe. Moreover, flows with the shortest RTT gain an additional advantage.

In large buffer configurations, all aspects of fairness are significantly disrupted. Here, sender distribution becomes the primary determinant of throughput disparity.

BBR, on the other hand, maintains nearly ideal fairness under shallow buffer conditions. Although shorter-RTT flows gain a slight advantage due to faster control loop updates, the disparity remains minor. As buffer size increases, fairness is no longer primarily influenced by RTT ratio, but by the relationship between RTT and buffer capacity. When inflight data becomes constrained by BBR’s internal congestion window limits, longer-RTT flows begin to dominate, and the degree of unfairness depends on sender group composition. Overall fairness decreases with more shorter-RTT flows, and STDR trends similarly. While RTS for dominating flows declines as the number of longer-RTT flows increases, the RTS for dominated flows shows a nonlinear pattern, peaking when sender groups are evenly split.

Although there is no single optimal buffer size for all network conditions, our findings show that excessively large buffers can be detrimental. Smaller buffers can significantly improve RTT fairness without compromising throughput. Notably, BBR exhibits a complex, buffer-sensitive fairness behavior, shaped by interactions between queuing depth and sender composition. Our results suggest that RTT fairness can be passively improved, without protocol modifications, through static buffer sizing or active queue management. These findings have important implications for network design, particularly in environments where latency sensitivity and fairness are critical, such as video conferencing, cloud gaming, or real-time communication systems. Network operators should carefully tune buffer sizing strategies to balance utilization and fairness across heterogeneous traffic.

Limitations and Future Work

This study employed Mininet emulation with a fixed dumbbell topology, loss-free links, and deterministic traffic. While this setup provides a controlled environment for isolating the effects of buffer sizing and RTT asymmetry, it does not capture all the dynamics of operational networks, such as wireless variability, random losses, cross-traffic bursts, diverse buffer management strategies, or heterogeneous link rates. Moreover, only Cubic and BBR were evaluated, and results were reported for a bounded set of buffer sizes and RTTs.

Future work could extend the evaluation to additional TCP variants, particularly BBRv2 and BBRv3, as well as mixed deployments where different TCP versions coexist. The traffic model could also be broadened to include short flows, mixed TCP/UDP traffic, and realistic flow arrivals and departures. Beyond traffic, further topological variations, such as wireless segments, lossy paths, or asymmetric links, should be explored. Finally, the analysis with three independent variables highlighted the need for richer statistical methods to capture nonlinear interactions and between-run variability better.

## Figures and Tables

**Table 1:** Comparison of mean values for overall fairness (JI), intra-group fairness (JI(G1), JI(G2)), average throughput (AvgThr) [Mbps], and average throughput [Mbps] for shorter-RTT and longer-RTT groups across different buffer categories (TCP Cubic).

Buffer Category	JI (Overall)	JI (G1)	JI (G2)	AvgThr	Thr (Short)	Thr (Long)
Small: <0.5 BDP	0.85	1.00	0.99	76	116	43
Medium: 0.5–2 BDP	0.95	0.98	0.98	96	114	77
Large: >2 BDP	0.88	0.97	0.95	97	129	74
All	0.90	0.98	0.97	92	120	67

**Table 2:** Comparison of mean values for overall fairness (JI), intra-group fairness (JI(G1), JI(G2)), average throughput (AvgThr) [Mbps], and average throughput [Mbps] for shorter-RTT and longer-RTT groups across different buffer categories (TCP BBR).

Buffer Category	JI (Overall)	JI (G1)	JI (G2)	AvgThr	Thr (Short)	Thr (Long)
Small: <0.5 BDP	0.99	1.00	1.00	96	99	95
Medium: 0.5–2 BDP	0.80	1.00	0.99	95	60	166
Large: >2 BDP	0.60	0.99	0.99	95	25	244
All	0.78	0.99	0.99	96	57	177

**Table 3:** Summary of best conditions for fairness in TCP Cubic and TCP BBR.

Algorithm	Buffer Size (Relative to BDP)	RTT Ratio Effect	Sender Distribution Effect	Best Fairness Conditions
Cubic	Medium (1–2 BDP)	High RTT ratio degrades fairness; small ratios yield best results	Weakly affected by sender distribution (except at extremes)	Medium buffers, balanced RTTs
BBR	Shallow (<0.5 BDP)	Less dependent on RTT ratio; fairness tied to buffer–RTT relation	Sensitive to imbalance when flows are congestion-window capped	Shallow buffers, ensuring inflight is constrained across RTTs

## Data Availability

The original data presented in the study are openly available in GitLab at https://gitlab.com/agnieszka.brachman/less-is-fair (accessed on 3 August 2025).

## Abbreviations

The following abbreviations are used in this manuscript:

AQM	Active Queue Management
BBR	Bottleneck Bandwidth and Round-trip propagation time
BDP	Bandwidth Delay Product
CC	Congestion Control
RTS	Relative Throughput Share
RTT	Round Trip Time
STDR	Symmetric Throughput Difference Ratio
TCP	Transmission Control Protocol

## References

[1] Cerf V., Dalal Y., Sunshine C. (1974). Specification of Internet Transmission Control Program RFC 675. https://datatracker.ietf.org/doc/html/rfc675.

[2] Kleinrock L. Power and deterministic rules of thumb for probabilistic problems in computer communications. Proceedings of the ICC 1979, International Conference on Communications.

[3] Jaffe J. (1981). Flow control power is nondecentralizable. IEEE Trans. Commun..

[4] Ha S., Rhee I., Xu L. (2008). CUBIC: A new TCP-friendly high-speed TCP variant. SIGOPS Oper. Syst. Rev..

[5] Cardwell N., Cheng Y., Gunn C.S., Yeganeh S.H., Swett I., Iyengar J., Vasiliev V., Jacobson V. BBR congestion control. Proceedings of the IETF 97th Meeting, Internet Engineering Task Force.

[6] Scholz D., Jaeger B., Schwaighofer L., Raumer D., Geyer F., Carle G. Towards a Deeper Understanding of TCP BBR Congestion Control. Proceedings of the 2018 IFIP Networking Conference (IFIP Networking) and Workshops.

[7] Hock M., Bless R., Zitterbart M. Experimental Evaluation of BBR Congestion Control. Proceedings of the 2017 IEEE 25th International Conference on Network Protocols (ICNP).

[8] Duke M., Fairhurst G. (2025). Specifying New Congestion Control Algorithms. RFC 9743. https://datatracker.ietf.org/doc/html/rfc9743.

[9] Brown P. Resource Sharing of TCP Connections with Different Round Trip Times. Proceedings of the IEEE INFOCOM 2000, Conference on Computer Communications, Nineteenth Annual Joint Conference of the IEEE Computer and Communications Societies (Cat. No.00CH37064).

[10] Gomez J., Kfoury E.F., Crichigno J., Srivastava G. (2024). Evaluating TCP BBRv3 Performance in Wired Broadband Networks. Comput. Commun..

[11] Kfoury E.F., Gomez J., Crichigno J., Bou-Harb E. (2020). An Emulation-Based Evaluation of TCP BBRv2 Alpha for Wired Broadband. Comput. Commun..

[12] Baker F., Fairhurst G. (2015). IETF Recommendations Regarding Active Queue Management. RFC 7567. https://datatracker.ietf.org/doc/html/rfc7567.

[13] Pan R., Natarajan P., Baker F., White G. (2017). Proportional Integral Controller Enhanced (PIE): A Lightweight Control Scheme to Address the Bufferbloat Problem. RFC 8033. https://datatracker.ietf.org/doc/html/rfc8033.

[14] Nichols K., Jacobson V., McGregor A., Iyengar J. (2018). Controlled Delay Active Queue Management. RFC 8289. https://datatracker.ietf.org/doc/html/rfc8289.

[15] Spang B., Arslan S., McKeown N. (2021). Updating the theory of buffer sizing. Perform. Eval..

[16] Xu L., Ha S., Rhee I., Goel V., Eggert L. (2023). CUBIC for Fast and Long-Distance Networks. Request for Comments RFC 9438, Internet Engineering Task Force. https://www.rfc-editor.org/info/rfc9438.

[17] Iyengar J., Thomson M. (2021). QUIC: A UDP-Based Multiplexed and Secure Transport. RFC 9000. https://datatracker.ietf.org/doc/html/rfc9000.

[18] Stewart R.R., Tüxen M., Nielsen K. (2022). Stream Control Transmission Protocol. RFC 9260. https://datatracker.ietf.org/doc/html/rfc9260.

[19] Kozu T., Akiyama Y., Yamaguchi S. Improving RTT Fairness on CUBIC TCP. Proceedings of the 2013 First International Symposium on Computing and Networking.

[20] Nemoto Y., Ogura K., Katto J. An adaptive TCP congestion control having RTT-fairness and inter-protocol friendliness. Proceedings of the 2013 IEEE 10th Consumer Communications and Networking Conference (CCNC).

[21] Appenzeller G., Keslassy I., McKeown N. Sizing router buffers. Proceedings of the 2004 Conference on Applications, Technologies, Architectures, and Protocols for Computer Communications.

[22] Dhamdhere A., Jiang H., Dovrolis C. Buffer sizing for congested Internet links. Proceedings of the IEEE 24th Annual Joint Conference of the IEEE Computer and Communications Societies..

[23] Enachescu M., Ganjali Y., Goel A., McKeown N., Roughgarden T. (2005). Part III: Routers with very small buffers. SIGCOMM Comput. Commun. Rev..

[24] Vishwanath A., Sivaraman V., Thottan M. (2009). Perspectives on router buffer sizing: Recent results and open problems. SIGCOMM Comput. Commun. Rev..

[25] Pan W., Tan H., Li X., Li X. (2021). Improved RTT Fairness of BBR Congestion Control Algorithm Based on Adaptive Congestion Window. Electronics.

[26] Cao Y., Jain A., Sharma K., Balasubramanian A., Gandhi A. When to Use and When Not to Use BBR: An Empirical Analysis and Evaluation Study. Proceedings of the Internet Measurement Conference.

[27] Cardwell N., Cheng Y., Yeganeh S.H., Swett I., Vasiliev V., Jha P., Seung Y., Mathis M., Jacobson V. BBRv2: A model-based congestion control. Proceedings of the IETF 104th Meeting, Internet Engineering Task Force.

[28] Song Y.J., Kim G.H., Mahmud I., Seo W.K., Cho Y.Z. (2021). Understanding of BBRv2: Evaluation and Comparison with BBRv1 Congestion Control Algorithm. IEEE Access.

[29] Cardwell N., Cheng Y., Yang K., Morley D., Yeganeh S.H., Jha P., Seung Y., Jacobson V., Swett I., Wu B. BBRv3: Algorithm Bug Fixes and Public Internet Deployment. Proceedings of the IETF 117th Meeting, Internet Engineering Task Force.

[30] Zeynali D., Weyulu E.N., Fathalli S., Chandrasekaran B., Feldmann A., Richter P., Bajpai V., Carisimo E. (2024). Promises and Potential of BBRv3. Proceedings of the Passive and Active Measurement.

[31] Ma S., Jiang J., Wang W., Li B. (2017). Fairness of Congestion-Based Congestion Control: Experimental Evaluation and Analysis. arxiv.

[32] Yang M., Yang P., Wen C., Liu Q., Luo J., Yu L. Adaptive-BBR: Fine-Grained Congestion Control with Improved Fairness and Low Latency. Proceedings of the 2019 IEEE Wireless Communications and Networking Conference (WCNC).

[33] Pan W., Li X., Tan H., Xu J., Li X. (2021). Improvement of RTT Fairness Problem in BBR Congestion Control Algorithm by Gamma Correction. Sensors.

[34] Yang S., Tang Y., Pan W., Wang H., Rong D., Zhang Z. (2023). Optimization of BBR Congestion Control Algorithm Based on Pacing Gain Model. Sensors.

[35] Han Z., Hasegawa G. (2025). Overcoming Fairness and Latency Challenges in BBR With an Adaptive Delay Detection. IEEE Access.

[36] Kim G.H., Cho Y.Z. (2019). Delay-Aware BBR Congestion Control Algorithm for RTT Fairness Improvement. IEEE Access.

[37] Zheng S., Liu J., Yan X., Xing Z., Di X., Qi H. (2024). BBR-R: Improving BBR Performance in Multi-Flow Competition Scenarios. Comput. Netw..

[38] Wang M., Zhang X., Jing F., Gao M. (2025). Ad-BBR: Enhancing Round-Trip Time Fairness and Transmission Stability in TCP-BBR. Future Internet.

[39] Kim G.H., Song Y.J., Mahmud I., Cho Y.Z. Enhanced BBR Congestion Control Algorithm for Improving RTT Fairness. Proceedings of the 2019 Eleventh International Conference on Ubiquitous and Future Networks (ICUFN).

[40] Njogu C.K., Yang W., Njogu H.W., Bosire A. (2023). BBR-with Enhanced Fairness (BBR-EFRA): A New Enhanced RTT Fairness for BBR Congestion Control Algorithm. Comput. Commun..

[41] Tao Y., Jiang J., Ma S., Wang L., Wang W., Li B. Unraveling the RTT-fairness Problem for BBR: A Queueing Model. Proceedings of the 2018 IEEE Global Communications Conference (GLOBECOM).

[42] tc-netem(8)-Linux Manual Page—man7.org. https://man7.org/linux/man-pages/man8/tc-netem.8.html.

[43] tc(8) - Linux Manual Page — man7.org. https://man7.org/linux/man-pages/man8/tc.8.html.

[44] Gueant V. iPerf—The TCP, UDP and SCTP Network Bandwidth Measurement Tool—iperf.fr. https://iperf.fr/.

[45] ss(8)-Linux Manual Page—man7.org. https://man7.org/linux/man-pages/man8/ss.8.html.

[46] Floyd S. (2008). Metrics for the Evaluation of Congestion Control Mechanisms. RFC 5166. https://datatracker.ietf.org/doc/html/rfc5166.

[47] Piotrowska A. GitLab Data Repository. https://gitlab.com/agnieszka.brachman/less-is-fair.

